# How relatedness between mates influences reproductive success: An experimental analysis of self‐fertilization and biparental inbreeding in a marine bryozoan

**DOI:** 10.1002/ece3.5636

**Published:** 2019-09-05

**Authors:** Scott C. Burgess, Lisa Sander, Marília Bueno

**Affiliations:** ^1^ Department of Biological Science Florida State University Tallahassee FL USA; ^2^ Present address: Departamento de Biologia Animal Instituto de Biologia Universidade Estadual de Campinas – UNICAMP Campinas Brazil

**Keywords:** bryozoan, *Bugula neritina*, dispersal, hermaphrodite, population genetics, self‐incompatibility

## Abstract

Kin associations increase the potential for inbreeding. The potential for inbreeding does not, however, make inbreeding inevitable. Numerous factors influence whether inbreeding preference, avoidance, or tolerance evolves, and, in hermaphrodites where both self‐fertilization and biparental inbreeding are possible, it remains particularly difficult to predict how selection acts on the overall inbreeding strategy, and to distinguish the type of inbreeding when making inferences from genetic markers. Therefore, we undertook an empirical analysis on an understudied type of mating system (spermcast mating in the marine bryozoan, *Bugula neritina*) that provides numerous opportunities for inbreeding preference, avoidance, and tolerance. We created experimental crosses, containing three generations from two populations to estimate how parental reproductive success varies across parental relatedness, ranging from self, siblings, and nonsiblings from within the same population. We found that the production of viable selfed offspring was extremely rare (only one colony produced three selfed offspring) and biparental inbreeding more common. Paternity analysis using 16 microsatellite markers confirmed outcrossing. The production of juveniles was lower for sib mating compared with nonsib mating. We found little evidence for consistent inbreeding, in terms of nonrandom mating, in adult samples collected from three populations, using multiple population genetic inferences. Our results suggest several testable hypotheses that potentially explain the overall mating and dispersal strategy in this species, including early inbreeding depression, inbreeding avoidance through cryptic mate choice, and differential dispersal distances of sperm and larvae.

## Graphical abstract

Our goal in this manuscript was to (a) experimentally assess how reproductive success depends on the relatedness of mates, including self, siblings, and nonsiblings within the same population and (b) to estimate inbreeding in natural adult populations using genetic markers. We found that reproductive success was lowest for self‐mating and highest for nonsib mating, and there was little evidence for consistent inbreeding in the field. Our results suggest that inbreeding depression, mate choice, and differential dispersal of sperm and larvae, could all potentially determine the amount of inbreeding and the overall mating and dispersal strategy in this species, and others like it

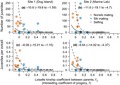

## 1 INTRODUCTION

In many species of plants and animals, organisms settle and breed near kin. Such kin associations increase the potential for inbreeding (mating with relatives; Wright, 1943). Although inbreeding often leads to varying degrees of inbreeding depression (the reduced fitness of inbred vs. noninbred progeny; Charlesworth & Charlesworth, 1999; Keller & Waller, 2002), it is still far from obvious how much inbreeding should and does actually occur (Addison & Hart, 2005; Carlon, 1999; Goodwillie, Kalisz, & Eckert, 2005; Knowlton & Jackson, 1993; Reid et al., 2015; Shields, 1982). Numerous factors influence whether individuals actively avoid or prefer mating with kin, or simply tolerate inbreeding depression because of the costs of avoiding it (Szulkin, Stopher, Pemberton, & Reid, 2013). Therefore, understanding the frequency and consequences of mating with kin is important for understanding the evolution of mating strategies and dispersal (Auld & Rubio de Casas, 2013; Cheptou & Massol, 2009; Grosberg, 1987; Knowlton & Jackson, 1993; Ravigné, Olivieri, Martinez, & Rousset, 2006; Shields, 1982), and their important effects on genetic variation, life history evolution, and responses to population decline (Charlesworth & Charlesworth, 1995; Hartfield, Bataillon, & Glémin, 2017; Lande & Porcher, 2015; Ryland & Bishop, 1993).

Many plants and clonal marine invertebrates with short dispersal potential are also hermaphrodites with the potential for self‐fertilization (Carlon, 1999; Jarne & Auld, 2006). As a result, inbreeding can occur from both self‐fertilization and biparental inbreeding (nonself matings with close relatives). However, most analyses of inbreeding in hermaphrodites focus on the dichotomy between self‐fertilization and outcrossing (though see Griffin & Eckert, 2003; Grosberg, 1987; Hoare & Hughes, 2001; Kelly & Willis, 2002), in an attempt to explain why both types of reproduction are common (known as mixed mating; Goodwillie et al., 2005; Jarne & Charlesworth, 1993). More recent analyses on biparental inbreeding have focused on animals with separate sexes to explain inbreeding avoidance (where mates are less related than expected under random mating), preference (where mates are more related than expected under random mating), or tolerance (where inbreeding occurs to the degree expected from random mating; Duthie & Reid, 2016; Szulkin et al., 2013). With both types of inbreeding possible, predictions for how selection acts on inbreeding become more complicated than when considering self‐fertilization and biparental inbreeding separately (Duthie & Reid, 2015; Szulkin et al., 2013). For example, the inclusive fitness benefits of inbreeding (which include the transmission advantage of selfing, Fisher, 1941) decline as the degree of relatedness between mates declines (Kokko & Ots, 2006). Self‐fertilization provides reproductive assurance (Lloyd & Schoen, 1992) that is not guaranteed with biparental inbreeding. As a result, dispersal, mate limitation, and mate choice will have differential effects on the overall system of inbreeding that is difficult to predict in any one species at present (Cheptou & Massol, 2009; Duthie & Reid, 2016; Porcher & Lande, 2016). Furthermore, the presence of biparental inbreeding in self‐compatible hermaphrodites leads to inflated estimates of the selfing rate and is difficult to distinguish from self‐fertilization, when making inferences from genetic markers (Ritland, 2002).

The fitness effects of biparental inbreeding in animals with separate sexes are also expected to be sex‐specific (Parker, 2006; Szulkin et al., 2013; Waser, Austad, & Keane, 1986), but sex‐specific effects of self‐fertilization and biparental inbreeding in hermaphrodites have received much less attention (though see, for example, Carr & Dudash, 1996; Hughes, Wright, Carvalho, & Hutchinson, 2009; Janicke, Vellnow, Sarda, & David, 2013; Rausher & Chang, 1999; Willis, 1999). Nonetheless, sex‐specific effects of inbreeding in hermaphrodites are also possible and, like in gonochoristic animals, have potential to create sexually antagonistic selection over whether to avoid or prefer inbreeding (Kokko & Ots, 2006; Parker, 2006). In hermaphrodites, such antagonism would manifest as male components of fitness being differentially affected by inbreeding or inbreeding avoidance compared with female components of fitness through intra‐ or interlocus conflict dynamics (Carr & Dudash, 1996; Charnov, 1979; Janicke et al., 2013; Schärer, Janicke, & Ramm, 2015; Willis, 1999). Typically, when reproductive success is limited by egg production in females and mate availability in males, female components of fitness benefit less from the inclusive fitness benefits of inbreeding than males components of fitness, where inbreeding could increase the individual's own reproductive success (Kokko & Ots, 2006; Parker, 2006; Perrin & Mazalov, 2000; Waser et al., 1986). In hermaphrodites, sex‐specific inbreeding depression is expected to favor a stable mixed‐mating strategy if outcrossing occurs with unrelated individuals (Janicke et al., 2013; Rausher & Chang, 1999). With the capacity for mate choice to control the outcome of any sexual conflict, and with sufficiently weak inbreeding depression, biparental inbreeding can favor inbreeding preference in females in gonochoristic animals if females control mate decisions, but the same is not true for males (Duthie & Reid, 2016).

Finally, the capacity to avoid or prefer inbreeding will also depend on the ability of individuals to discriminate among relatives and unrelated conspecifics, which will depend on the degree and range of relatedness between potential mates, the genetic basis of any self‐incompatibility mechanism, and the structural characteristics of the mating system that determine the mode and timing of gamete transfer (Duthie & Reid, 2016; Eckert, 2011; Jarne & Charlesworth, 1993; Kelly & Willis, 2002; Lloyd & Schoen, 1992). For example, copulation allows greater potential for mate choice to avoid or prefer biparental inbreeding than do mating systems where pollen or sperm are shed. Many animals in the sea shed sperm into the water. In these cases, fertilization occurs internally after sperm capture in “spermcasting” species (Bishop & Pemberton, 2006) or externally in “broadcasting” species that also release eggs into the water (Levitan & Petersen, 1995). Spermcast mating tends to be associated with brooding of embryos, the release of short dispersing larvae, and a high potential for inbreeding (Knowlton & Jackson, 1993). However, and unlike in plants requiring pollinators, ciliary‐driven feeding currents used for suspension feeding also facilitate active capture, concentration, storage, and potential selectivity of waterborne sperm from dilute suspension (Hughes, Manriquez, & Bishop, 2002; Pemberton, Hughes, Manríquez, & Bishop, 2003). A spermcast mode of gamete transfer suggests less male gamete limitation, high competition among males (Yund & McCartney, 1994), and increased female control over paternity to promote or avoid inbreeding than is typical in plants, or in a broadcasting mode of gamete transfer in other marine invertebrates (Addison & Hart, 2005; Charnov, 1979).

Clearly, predicting the frequency of inbreeding when inbreeding is likely is complicated because evolution of the overall mating system is mediated by many factors. As a result, it remains difficult to predict the overall inbreeding strategy in hermaphrodites where both selfing and biparental inbreeding are possible (Porcher & Lande, 2016; Uyenoyama, 1986). It is therefore important to empirically characterize how reproductive success varies across different values of relatedness between parents in species with mating systems that provide different opportunities for, and cost of, inbreeding compared with those more commonly studied. Accordingly, our study species was the marine bryozoan, or “moss animal,” *Bugula neritina*—a sessile spermcaster, with the capacity to self‐fertilize. Like many sessile clonal benthic marine invertebrates (Jackson, 1986), most larvae settle within a meter of the maternal colony (S. C. Burgess, in preparation), increasing the potential for biparental inbreeding. The distribution of adult *B. neritina* in fairly continuous seagrass habitats is highly patchy in space and consistent over time; individuals are consistently absent from suitable sites <100 m away, suggesting dispersal limitation within this scale (Keough & Chernoff, 1987). Nearest neighbor distances are typically in the order of centimeters (Keough, 1989). There is substantial variation in genetic relatedness across scales of centimeters to meters (S. C. Burgess, in preparation), creating opportunities for inbreeding preference or avoidance to evolve. Furthermore, in populations of *B. neritina* in California, larvae appear to actively settle closer to kin than to nonkin (Keough, 1984), although we have not found evidence for this in our populations from the northern Gulf of Mexico (see also Raimondi & Keough, 1990).

Our goal was to (a) experimentally assess how reproductive success depends on the relatedness of mates, ranging from self, siblings, to nonsiblings from within the same population and (b) estimate inbreeding in natural adult populations using genetic markers. We found that biparental inbreeding was more common than selfing, reproductive success was highest for nonsib mating, and there was little evidence for inbreeding in the field. We were able to culture *B. neritina* from larvae to reproductive maturity and confirm paternity using 16 newly developed microsatellite markers (detailed in Appendix S1). In this species, investment into female reproduction is easily quantified by the presence and number of external brood chambers (ovicells). Reproductive success is estimated by the number of larvae that are released and successfully metamorphose.

## 2 METHODS

### 2.1 Study species

*Bugula neritina* (Linnaeus, 1978) is an arborescent bryozoan from the Phylum Bryozoa (Class: Gymnolaemata; Order: Cheilostomata). There are about 5,900 described living species of bryozoans and up to 11,100 expert‐based estimated number of species (Appeltans et al., 2012; Brusca, Moore, & Shuster, 2016). An individual is a colony that grows through the addition of asexually produced zooids. Each zooid contains all, or at least most, of the nutritive, reproductive, and other organs needed to be self‐supporting (called autozooids; Silén, 1977). Autozooids are physiologically connected through a conducting system of hollow epithelial tissue (the funiculus) and communication pores in the interzooid walls. All bryozoans are hermaphroditic at the colony level, but the patterns of hermaphroditism within colonies are complex and variable (Ostrovsky, 2013). In *B. neritina*, Ostrovsky (2013) described the presence of sterile and sexual zooids. Sexual zooids are female or male with no morphological distinctions. Male zooids are located proximally (nearer to the point of attachment) than female zooids (Ostrovsky, 2013, p. 7), such that colonies are simultaneous hermaphrodites and capable of self‐fertilization through uptake of sperm from male zooids on the same colony. Female zooids contain a brood chamber (called an ovicell) on the outside of the zooid. Ovicells are noted to develop only when a mature egg is present in the coelom (Ostrovsky, 2013; Ström, 1977). Colonies do not begin to produce ovicells until about three weeks or six (total) bifurcations (at 23–25°C, unpublished data). Presumably, colonies are first sterile (though could still possibly store sperm from conspecifics; Hughes et al., 2002) and then contain male zooids, then male and female zooids, as more zooids are added. A closely related species (*Bugula flabellata*) was described as having hermaphroditic zooids (Dyrynda & Ryland, 1982), and it is not known whether individual zooids in *B. neritina* can also be simultaneously hermaphroditic or change from male to female, or vice versa, as the zooid ages. The fertilization process for *B. nertina* specifically has not been described, but observations on numerous similar species have led to the consensus that, in all bryozoans, individual spermatozoa are released through a terminal pore in the tips of the lophophore tentacles (Bullivant, 1967; Silén, 1966, 1972; Temkin, 1994). Eggs are retained, and fertilization occurs inside the maternal zooid from sperm acquired from the water (spermcast mating; Bishop & Pemberton, 2006). Intrazooid self‐fertilization is considered unlikely (Bullivant, 1967; Silén, 1966). The fertilized oocyte is transferred into the ovicell on the outside of the female zooid, where it increases in volume ~500‐fold over ~7 days (Woollacott & Zimmer, 1975) and develops into a coronate larva (~250–350 μm diameter). Larval development is supported by extraembryonic nutrition through a placenta‐like structure (Ostrovsky, 2013; Woollacott & Zimmer, 1975). Black embryos are clearly visible inside ovicells, which are otherwise white. Embryos can be aborted during this brooding period in similar species (Hunter & Hughes, 1993). An ovicell broods a single embryo at a time, though it is possible that multiple larvae can be produced in sequence from stored sperm (Ostrovsky, 2013; Ström, 1977). The possibility of multiple larvae being produced in sequence, and that the time of fertilization and stage of brooding could differ among zooids within a colony, makes it unclear whether a count of the rate of ovicell occupancy provides a reliable estimate of the fertilization rate, even though ovicells develop when eggs are mature and black embryos are clearly visible inside ovicells. Competent, ciliated, nonfeeding larvae settle within minutes to hours once released from the ovicell (Keough, 1989). Most larvae settle within a meter of the maternal colony (unpublished data), but the distributions of sperm dispersal distances are currently unknown in this species. Prolonged larval duration (more than several hours) results in reduced postsettlement survival from carryover effects of delayed metamorphosis that also reduces successful dispersal distances (Burgess, Bode, & Marshall, 2013; Burgess, Treml, & Marshall, 2012; Wendt, 1998).

### 2.2 Sample collection

Sexually mature colonies (those large enough to contain ovicells) were collected from three sites near the Florida State University Coastal and Marine Laboratory in the northern Gulf of Mexico (St. Teresa, FL). Sites were separated by between 10 and 25 km. At Site 1 (Dog Island) and Site 2 (Marine Lab) (these sites are separated by about 10 km), *Bugula* primarily lives attached to seagrass (*Syringodium filiforme*, *Thalassia testudinum*, and *Halodule wrightii*) in 0.5–2 m water depth (Keough & Chernoff, 1987). Colonies from these sites were collected on snorkel on 30 October 2017 and used in the breeding experiment. At Site 3 (One More Time), *Bugula* lives attached to an artificial reef (22 m shrimp boat scuttled in 1992) in ~12 m water depth. Colonies from this site were collected on SCUBA on 15 March and 2 April 2018.

A total of 30, 10, and 34 colonies were collected and genotyped from Site 1 (Dog Island), Site 2 (Marine Lab), and Site 3 (One More Time), respectively. Larvae from four colonies from Site 1 (Dog Island) and four colonies from Site 2 (Marine Lab) were used as parents in the experimental crosses (see Section 2.4).

### 2.3 DNA analysis

All samples were amplified at 16 microsatellite loci. Details on the isolation and characterization of these microsatellite markers are provided in the Supplement. Total genomic DNA was extracted from ~30 mg of tissue using OMEGA Bio‐Tek E.Z.N.A^®^ Tissue DNA Kit following the manufacturer's protocol. DNA quality and quantity were assessed by spectroscopy (NanoDrop 1,000). Polymerase chain reaction (PCR) was performed in an 8 µl volume using 1–10 ng of template DNA, 0.025 µM forward primer, 0.1 µM reverse primer, 0.1 µM fluorescent primer, 0.01% BSA, and 2X GoTaq^®^ Colorless Master Mix. All forward primers were tailed at the 5′ end with one of the following universal tails: (M13) 5′‐TGT AAA ACG ACG GCC AGT‐3′, (C) 5′‐CAG GAC CAG GCT ACC GTG‐3′, or (D) 5′‐CGG AGA GCC GAG AGG TG‐3′. One of the following fluorescent dyes was incorporated into each of the amplicons via a second step PCR containing an oligo homologous to the previously described tails: FAM, HEX, NED, PET, or VIC. PCR thermal cycling parameters were as follows: initial denaturation at 95° (5 min) followed by 8 cycles of 95° (30 s), 56° (30 s), 72° (45 s) and 30 cycles of 95° (30 s), 53° (30 s), 72° (45 s), and a final extension at 72° (15 min). For each sample, amplicons were pooled in approximately equal ratios with four amplicons per pool. Samples for fragment analysis contained 1.5 μl of pooled amplicons, 0.15 μl LIZ size standard, and 12 µl Hi‐Di formamide. Purified PCR products were separated on an Applied Biosystems 3730 Genetic Analyzer with Capillary Electrophoresis in the Biology Core Facility at Florida State University. Fragment sizes and polymorphism were assessed using the program Geneious v9.1.8, and all alleles were called manually.

To confirm our samples did not include cryptic species, we also sequenced and aligned 432 base pairs of the mitochondrial cytochrome oxidase c subunit I (COI) gene from the eight grandparents used in the breeding experiment. PCR amplification was carried out using the *B. neritina*‐specific primer pair BnCOIf (5′‐3′ sequence: ACAGCTCATGCATTTTTA) and BnCOIr (5′‐3′ sequence: CATTACGATCGGTTAGTAG) (Linneman, Paulus, Lim‐Fong, & Lopanik, 2014). Sequences from all eight grandparents were identical to each other and were identical to the Type S1 haplotype of Davidson and Haygood (1999) (GenBank accession numbers AF061432, AF061426) and the Type S (Shallow) haplotype of McGovern and Hellberg (2003) (GenBank accession number AY173425). This shallow‐water haplotype is the most common and widely distributed haplotype within the *B. neritina* cryptic species complex, and our finding is consistent with sampling in the same region by others (Davidson & Haygood, 1999; Fehlauer‐Ale et al., 2014; Mackie, Keough, & Christidis, 2006; McGovern & Hellberg, 2003).

### 2.4 Estimates of selfing and biparental inbreeding using experimental crosses

**Figure 1:**
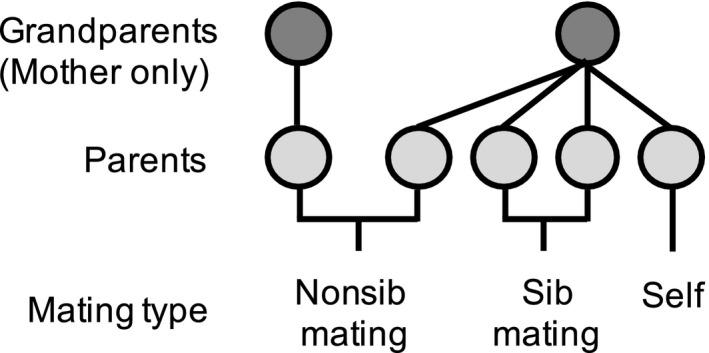
Structure of the experimental crosses used to estimate the relationship between parental relatedness and reproductive success. Larvae from eight colonies (grandmothers) randomly collected from two populations in the field (four colonies from each population) served as parents (*n* = 45; 4–7 from each “grandmother”). Parents were allocated to one of three mating type treatments, and their growth and offspring number were recorded. Individual parents served as both fathers and mothers

The colonies collected and genotyped from Site 1 (Dog Island, *n* = 30) and Site 2 (Marine Lab, *n* = 10) formed the grandparent (first) generation for the experimental crosses. Four randomly selected colonies from each site were selected as grandmothers. Larvae were collected from the eight grandmothers using a light treatment (Burgess et al., 2012) and allowed to settle and metamorphose on roughened, bio‐filmed, acetate sheets. These settlers formed the parental (second) generation, and 45 were chosen as parents for the breeding experiment (6–9 from each grandmother; Figure 1). The parental generation was then reared in environmental chambers for 43 days (Table 1) at 25°C using a 11:13 light:dark regime. Seawater (collected from the field and autoclaved) was changed every two, sometimes three, days and dosed with live phytoplankton cells (*Rhodomonas salina*) for food (at ~10^5^ cells per ml). Great care was taken to avoid sperm contamination during water changes. Up until 19 days, parents from known grandmothers were cultured individually in isolated aquaria (250 ml glass bowl) to prevent sperm contamination from other colonies as well as differences in colony size emerging from competition for food. At day 19 (when ovicells were first noticed, indicating the onset of female reproduction), the 45 parental colonies were placed into separate aquaria in one of three mating treatments (i.e., 4–7 siblings from each grandmother were split among the three treatments, so that each treatment contained 1–3 siblings):

1. In isolation (Self). This treatment estimated self‐fertilization in the absence of conspecific sperm.
2. With siblings from the same grandmaternal colony (Sib mating). This treatment estimated biparental inbreeding.
3. With parents originating from colonies collected from within the same site but separated in space by >10 m (Nonsib mating). This treatment estimated mating success between nonsibs within the same population.

**Table 1:** Schedule of events for the experimental crosses

Day	Events
0	Settlers (=parents) were obtained from field‐collected grandparents and reared in isolation. Grandparents sacrificed for genotyping
19	Parents allocated into one of four mating partner treatments. Colonies size ranged from 4 to 7 bifurcations. Only two colonies had ovicells present
33	Parents placed back in isolated aquaria
37	37 colonies (out of 45) had ovicells present. 19 colonies had produced settlers
43	Number of settlers (=juvenile offspring summed over 10 days), ovicells, and wet mass per colony recorded. Parents sacrificed for genotyping. 24 colonies produced settlers
51	Settlers (=juvenile offspring) sacrificed for genotyping. No mortality observed

Fourteen days after exposure to these treatments, during which time colonies were able to exchange waterborne sperm, each parental colony was transferred back to isolated aquaria. Roughened, bio‐filmed, acetate sheets were then floated on the surface of the water, to which all released larvae from a known mother settled and attached. These settlers then formed the offspring (third) generation. The total number of settlers was recorded and summed over a ten‐day period after being transferred back to isolated aquaria (Table 1), after which the number of settlers declined. Acetate sheets were exchanged as needed. At this point, parent colonies were sacrificed. Wet mass (in grams) was measured by gently patting the colony with a paper towel to remove excess water and weighing on a bench‐top analytic balance. The number of ovicells per colony was counted under a dissecting microscope. Eight‐day‐old settlers were genotyped to confirm paternity from all mothers that produced offspring (24 out of 45, or 53% of, mothers in total; there was no settler mortality): Three offspring were genotyped from 17 mothers, and 2–23 offspring were genotyped from the remaining seven mothers. In sum, 74 colonies (from three sites) from the grandmother generation (eight serving as grandparents in the experimental crosses), 45 colonies from the parent generation, and 158 colonies from the offspring generation were genotyped (277 individuals in total).

### 2.5 Paternity analysis of the experimental crosses

We conducted a paternity analysis of known maternal broods to check: (a) whether self‐fertilization occurred, which could still occur in the presence of conspecifics, and (b) that there was no sperm contamination by inadvertently transferring drops of water containing sperm between culture vessels. Paternity assignment was based on consensus from two programs: cervus v3.0.7 (Kalinowski, Taper, & Marhsall, 2007) and colony v2.0.6.4 (Jones & Wang, 2010). Both are based on likelihood methods, account for mistyping errors, and were conducted on the progeny array with known maternal–offspring relationships. In both programs, the “allele frequencies unknown” option was chosen, so were estimated from the dataset within which relationships were being inferred.

For cervus analysis, simulations were used to calculate strict (95%) and relaxed (80%) confidence levels for assignments, and were conducted on 10,000 offspring, 100% candidate fathers, and a 1% error rate. For colony analysis, input parameters were as follows: both sexes polygamous and monoecious (i.e., hermaphroditic), inbreeding present, diploid, two long runs of the full‐likelihood model, very high likelihood precision, and no updating of allele frequencies. Marker error rates and null allele frequencies were set at 0.001, and percent sampled candidate fathers set at 100%.

### 2.6 Statistical analyses of the experimental crosses

#### 2.6.1 Relationship categories

Generalized linear mixed effects models were used to estimate how colony wet mass (Gaussian), the number of juveniles (settlers; Poisson), and the number of juveniles per ovicell (or the proportion of ovicells that produced juveniles; binomial) for each parent differed among mating partner treatments. Grandmothers were modeled as a random effect. Since only one parent in the selfing treatment produced three settlers, this treatment was removed from the analyses except for the analysis on colony wet mass. Each site was analyzed separately for all analyses. Chi‐square likelihood ratio tests were used to determine whether the differences between treatment means were significantly different to zero. The calculated 95% confidence intervals on the fitted values include both the uncertainty in the fixed effect estimate (i.e., the treatment mean) and the random effect variance (i.e., the average variance among parents with different grandmothers). Analyses were performed in R v3.5.1 (R Core Team, 2018) using the lme4 package (v1.1‐21).

#### 2.6.2 Kinship coefficients between parents

The coefficient of kinship *f_i,j_* between two individuals *i* and *j* measures the probability that two homologous alleles, one chosen randomly from each individual, are identical by descent (IBD) (Blouin, 2003). An individual's inbreeding coefficient is the same as the coefficient of kinship between their parents (Crow & Kimura, 1970, p66‐69). We estimated kinship coefficients between parents used in the experimental crosses, as a measure of their offspring's inbreeding coefficient, using the 16 microsatellite markers. We used the program SPAGeDi v1.5 (Hardy & Vekemans, 2002) to calculate the Loiselle kinship coefficient (Loiselle, Sork, Nason, & Graham, 1995). The reference allele frequency was that calculated from the 40 colonies in the “grandparent” generation that were randomly collected from Site 1 (Dog Island) and Site 2 (Marine Lab) (eight of which were the grandmothers of the parents in the experimental crosses).

Generalized linear mixed effects models were used to estimate the effect of kinship on the number of juveniles (Poisson) and the proportion of ovicells that produced juveniles (binomial) in the experimental crosses. Since these variables were measured in both partners of a particular cross, where both parents have the same coefficient of kinship, each parental pair was modeled as a random effect.

### 2.7 Estimates of inbreeding from population genetic data

A total of 30, 10, and 34 colonies were genotyped from Dog Island, Marine Lab, and One More Time, respectively, using 16 microsatellite markers (see Section 2.3). We estimated the inbreeding rate in these 74 samples using multiple methods. For all methods, estimates of the inbreeding rate based on population genetic markers integrate the effects of both self‐fertilization and biparental inbreeding over several generations, as well as mortality from inbreeding depression prior to samples being collected from the field. The first method used the fixation index *F*_IS_, which is the proportionate reduction in heterozygosity due to inbreeding relative to the subpopulation as a whole (calculated in the program genodive v2.0b23, Meirmans & Van Tienderen, 2004). *F*_IS_ is related to the selfing rate, *s*, at equilibrium using the classic formula *s*(*F*_IS_) = 2*F*_IS_/*F*_IS_ + 1 (Hedrick & Cockerham, 1986). A drawback of the *F*_IS_ method is that factors other than selfing, such as null alleles, can also cause heterozygote deficiencies. Therefore, we also estimated the inbreeding rate based on the distributions of multilocus heterozygosity in the program Robust Multilocus Estimation of Selfing (RMES; David, Pujol, Viard, Castella, & Goudet, 2007). This program provides two methods (*g*_2_ and Maximum Likelihood; see David et al., 2007 for details) to estimate inbreeding, which are both independent of *F*_IS_, and which are considered more reliable. Since inbreeding also increases linkage disequilibrium, we also assessed multilocus linkage disequilibrium by calculating the index of association (*I*_A_) and the standardized index of association that accounts for the number of loci (${\bar{r}}_{d}$) using the *poppr* package in R (Kamvar, Tabima, & Grünwald, 2014). The null hypothesis tested was that alleles observed at different loci are not linked and alleles recombine freely into new genotypes. We also estimated individual inbreeding coefficients *f*, defined as the probability of identity by descent of two alleles at a locus in an individual (Keller & Waller, 2002; Wright, 1922). Individual inbreeding coefficients were estimated using the triadic likelihood estimator of Wang (2007) implemented in the program coancestry v1.0.1.8 (Wang, 2011). This method allows for prior inbreeding by estimating the full nine condensed identity‐by‐decent coefficients between two focal individuals (Wang, 2007).

## 3 RESULTS

### 3.1 Paternity analysis of the experimental crosses

Paternity assignment results from cervus and colony were in agreement and confirmed outcrossing, no sperm contamination, and no polyembryony (Craig, Slobodkin, Wray, & Biermann, 1997; Jenkins, Waeschenbach, Okamura, Hughes, & Bishop, 2017; Johnson, 2010). In both programs, when all 45 individuals in the parental generation were allowed to be candidate fathers, the most likely father for each offspring was the individual paired with the offspring's known mother, with one exception. For one offspring, both programs incorrectly assigned paternity to a sibling of the individual paired with this offspring's mother. This single assignment error was also confirmed by manual inspection of the genotypes, which revealed unique alleles in four loci for the assigned father only that were never represented in the offspring. Together, this suggests that the individual paired with the known mother in the experiment was the true father, rather than the father assigned by the programs. In all of the remaining 157 offspring, the most likely father was assigned paternity at the strictest confidence level (95%) in cervus, or with a probability of 1 in colony.

Three offspring produced from a single individual reared in isolation were confirmed as resulting from self‐fertilization (from the paternity analysis in both programs and through manual inspection of the genotypes). There was no other evidence for self‐fertilization.

Even though paternity assignments were robust using these markers, cervus identified five mismatches (three families at two loci) in known mother–offspring genotypes (Table S6). All five mismatches could be explained by the inheritance of a null allele (i.e., when offspring and known mother are homozygous for different alleles). The mistyping rate was zero in the remaining 14 loci (Table S6).

### 3.2 Estimates of selfing and biparental inbreeding using experimental crosses

#### 3.2.1 Growth and investment in female zooids

After 43 days growing in the laboratory, colony wet mass did not differ between the three types of crosses (Dog Island: *X* ^2^ = 2.8, *df* = 2, *p* = .25; Marine Lab: *X* ^2^ = 4.78, *df* = 2, *p* = .09). By this time, all colonies produced ovicells, except two colonies reared in isolation and one colony from a sib mating from Site 2 (Marine Lab; Table 2). The average number of ovicells per colony at Site 1 (Dog Island) was 440 (±74 *SE*) and at Site 2 (Marine Lab) was 437 (±80 *SE*).

**Table 2:** Number (percent) of experimental colonies that produced ovicells and settlers in the laboratory

Treatment	Alone		Sib mating		Nonsib mating	
	Present	Absent	Present	Absent	Present	Absent
Site 1 (Dog Island)						
Ovicells	4 (100%)	0	8 (100%)	0	8 (100%)	0
Settlers	0 (0%)	4	4 (50%)	4	8 (100%)	0
Site 2 (Marine Lab)						
Ovicells	7 (77%)	2	7 (87%)	1	8 (100%)	0
Settlers	1 (11%)	8	3 (37%)	5	8 (100%)	0

#### 3.2.2 Number of juveniles

**Figure 2:**
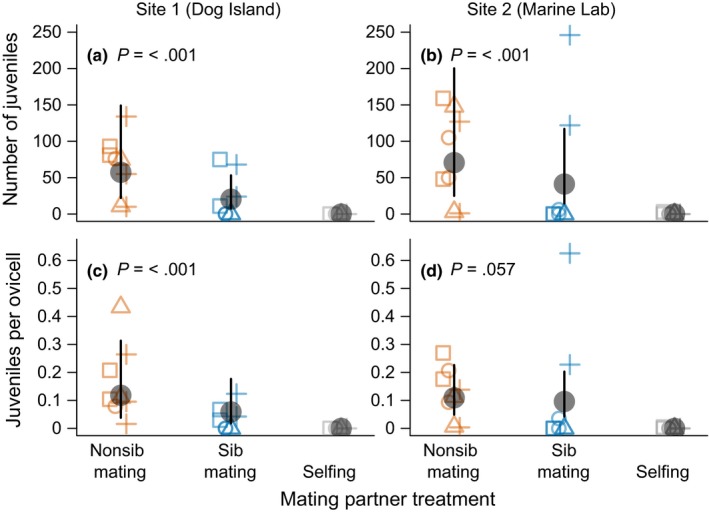
The relationship between parental relationship category (mating partner treatment) and reproductive success, in terms of the number of juveniles (settlers) produced per colony (a, b), and the number of viable juveniles (settlers) produced per ovicell per colony (c, d) in two populations (left and right panels). Mating partner treatments are described in Figure 1. Large black circles and vertical bars indicate the mean and 95% confidence intervals estimated from generalized linear mixed effects models, with grandmother ID as a random effect. Confidence intervals include both the uncertainty in the fitted mean and the random effect variance due to average differences among parents with different grandmothers. The selfing treatment was not included in the statistical models because only one colony produced three offspring in that treatment, so the *p*‐value (*p*) is the probability of obtaining the observed test statistic if there were no differences in the mean between sib matings and nonsib matings. Each point indicates a single parent and different symbols indicate parents from different grandmothers

In both populations, the number of juveniles was lower for sib matings compared with nonsib matings from the same population (Figure 2a,b). At Site 1 (Dog Island), sib matings produced only 35% (30–42, 95% confidence interval) of the number of offspring produced in nonsib matings. At Site 2 (Marine Lab), sib matings produced 58% (51–66, 95% confidence interval) of the number of offspring produced in nonsib matings. For matings between sibs and between nonsibs, the number of settlers produced positively covaried within each mating pair (*r*_spearman's_ = .83, *p* < .001).

#### 3.2.3 Juveniles per ovicell

Patterns in the proportion of juveniles per ovicell largely mirrored that for the total number of juveniles. At Site 1 (Dog Island), the proportion of juveniles per ovicell averaged 0.06 (0.02–0.18, 95% confidence interval) for sib matings and averaged 0.12 (0.04–0.30, 95% confidence interval) for nonsib matings. At Site 2 (Marine Lab), the proportion of juveniles per ovicell was 0.1 (0.04–0.20, 95% confidence interval) for sib matings and 0.11 (0.05–0.23, 95% confidence interval) for nonsib matings. The proportion of juveniles per ovicell for sib matings was driven by relatively high reproductive success from only a single pair of sibs (plus symbols in Figure 2b,d).

### 3.3 Kinship

**Figure 3:**
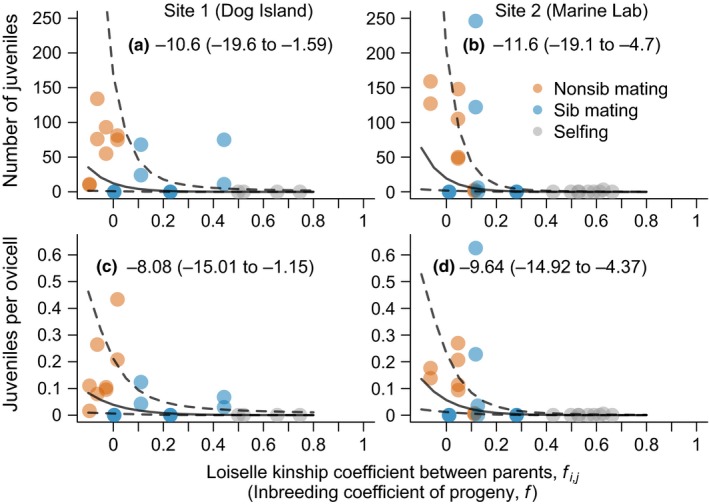
The relationship between the Loiselle kinship coefficient between parents, *f_i,j_* (=inbreeding coefficient of their offspring, *f*) and reproductive success, in terms of the number of viable juveniles (settlers) produced per colony (a, b), and the number of viable juveniles produced per ovicell (b, c) in two populations (left and right panels). Each point represents the reproductive success of an individual mother, colored by their relationship category. Lines show fitted means and 95% confidence intervals from generalized linear mixed effects models (Poisson in a, b, and binomial in c, d) where parental pairs (which have the same kinship coefficient) were modeled as a random effect. The slope coefficient and 95% confidence interval, on the scale of the link function, are presented

**Figure 4:**
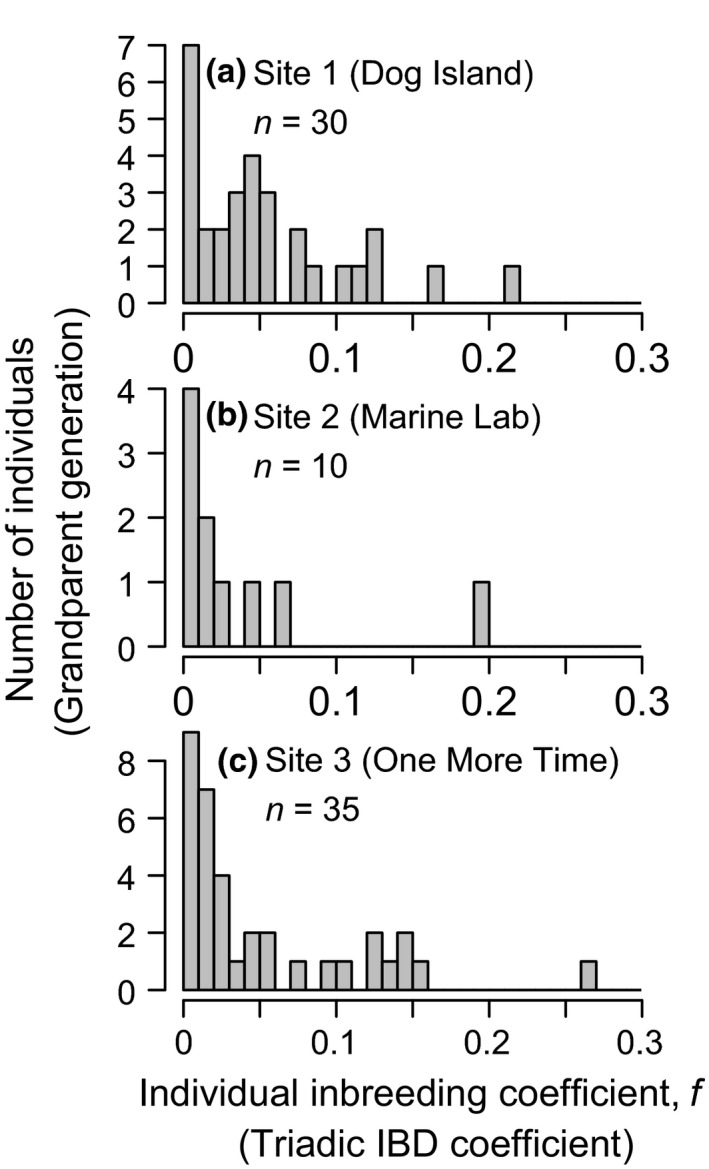
Individual inbreeding coefficients *f* (the probability of identity by descent [=IBD] of two alleles at a locus in an individual) in three populations (a–c). Coefficients were estimated using the triadic likelihood estimator (Triadic IBD coefficient) implemented in the program coancestry. This method allows for prior inbreeding when estimating IBD. The mean (95% confidence interval) inbreeding coefficient at each population was as follows: Site 1 = 0.05 (0–0.18); Site 2 = 0.04 (0–0.17); Site 3 = 0.05 (0–0.17). For reference, in an outbred population, the expected *f* for an individual with half‐sib parents is 0.125 and for full‐sib parents is 0.25

The number of juveniles, and the number of juveniles as a proportion of the number of ovicells, declined with increasing kinship between parents at both sites (Figure 3). As expected, siblings generally had higher kinship coefficients than nonsiblings, though kinship coefficients varied within relationship categories. This potentially reflects the presence of both full‐ and half‐sibs, individual variation in their recent history of inbreeding (e.g., Figure 4), stochastic differences in true IBD among loci, and the chance sharing of alleles that are identical by state (Blouin, 2003).

### 3.4 Estimates of inbreeding from population genetic data

Each individual colony (*n* = 74) randomly collected from across three populations in the field (i.e., the grandparent generation in the experimental crosses) had a unique multilocus genotype (i.e., there were no clones). For these adult colonies, there was no evidence for self‐fertilization or biparental inbreeding across three indices of inbreeding at the population level (Table 3). The estimated population selfing rate was never significantly different to zero. Across all three populations, the number of alleles per locus ranged from 3–17, and there was no evidence for significant deviations from Hardy–Weinberg equilibrium (Table S2–S4). There was no evidence for multilocus linkage disequilibrium in populations at Site 1 (Dog Island) and Site 2 (Marine Lab), but there was at Site 3 (One More Time; Table 4).

**Table 3:** Estimated rate of inbreeding (including selfing, *s*) using three multilocus methods: *F*_IS_, *g*_2_, and maximum likelihood (ML)

Population	*N*	*N*_g_	*L*	A	*H*_o_	*H*_e_	*F*_IS_ method		*g*_2_ method		ML method
							*F*_IS_ ($P_{F_{\text{IS}}=0}$)	*s*(*F*_IS_)	*g*_2_	*s*(*g*_2_) ($P_{g_{2}=0}$)	*s*(ML) [95% CI]
Site 1 (Dog Island)	30	30	16	8.25	0.73	0.75	0.020 (*p* = .15)	0.039	−0.008	0 (*p* = .890)	0 [0, 0.043]
Site 2 (Marine Lab)	10	10	16	6.38	0.78	0.76	−0.034 (*p* = .19)	−0.070	0.005	0.020 (*p* = .224)	0 [0, 0.103]
Site 3 (One More Time)	34	34	16	8.63	0.74	0.72	−0.028 (*p* = .12)	−0.068	0.001	0.004 (*p* = .388)	0 [0, 0.048]

Note

Samples from Site 1 (Dog Island) and Site 2 (Marine Lab) contained the grandparent generation used in the experimental crosses. For the ML method, 95% confidence intervals (CI) of *s* are given. *F*_IS_ was calculated from the program GenoDive using Weir & Cockerham's (1984) method. The selfing rate using the *F*_IS_ method was calculated as *s*(*F*_IS_) = (2*F*_IS_)/(*F*_IS_ + 1). The *g*_2_ and maximum likelihood (ML) method were calculated in the program RMES (Robust Multilocus Estimation of Selfing).

Abbreviations: A, average number of alleles per locus; *H*_e_, expected heterozygosity; *H*_o_, observed heterozygosity; *L*, number of microsatellite loci; *N*, number of individuals genotyped; *N*_g_, number of unique multilocus genotypes; *p*, *p*‐value.

**Table 4:** Estimates of multilocus linkage disequilibrium

Population	*N*	*N*_g_	*L*	*I*_A_	${\bar{r}}_{d}$	*p*
Site 1 (Dog Island)	30	30	16	0.141	.0094	.081
Site 2 (Marine Lab)	10	10	16	0.232	.0158	.153
Site 3 (One More Time)	34	34	16	0.21	.0141	.0193

Abbreviations: ${\bar{r}}_{d}$, standardized index of association that accounts for the number of loci; *I*_A_, index of association; *L*, number of microsatellite loci; *N*, number of individuals genotyped; *N*_g_, number of unique multilocus genotypes; *p*, the probability of the observed if there is no linkage among loci (based on 10,000 permutations).

The distribution of individual‐level inbreeding coefficients also showed an equally low mean level of inbreeding, but also revealed within population variation in an individual's history of inbreeding (Figure 4). A few individuals had inbreeding coefficients close to that expected if their parents were outbred half‐sibs (expected *f* = 0.125) and outbred full‐sibs (expected *f* = 0.25), but there was no evidence for selfing.

## 4 DISCUSSION

An important part of predicting the prevalence of inbreeding when inbreeding is likely to understand how reproductive success depends on the relatedness of mates. In this context, most analysis on hermaphrodites focus on the prevalence of selfing versus outcrossing (usually with unrelated mates), and the role of mate limitation and inbreeding depression (Goodwillie et al., 2005; Jarne & Auld, 2006; Jarne & Charlesworth, 1993). When dispersal and mating are localized, outcrossing can still result in mating with relatives (biparental inbreeding) (Griffin & Eckert, 2003; Grosberg, 1987; Hoare & Hughes, 2001; Kelly & Willis, 2002). The combination of self‐fertilization and biparental inbreeding makes it difficult to distinguish the type of inbreeding when making inferences from genetic markers, and it complicates predictions for how inbreeding avoidance, preference, or tolerance evolves, so requires studies that simultaneously manipulate both types of inbreeding. We focused on an understudied mating system (spermcast mating) that provides numerous opportunities for both inbreeding preference and avoidance, and exhibits important differences to more commonly studied mating systems in plants or copulation mating in animals. Unlike most studies in hermaphrodites, we performed experimental crosses to estimate both selfing and biparental inbreeding. We found that the production of viable selfed offspring was extremely rare (only one colony produced three selfed offspring) and biparental inbreeding more common. In both populations, the number of viable juveniles produced was higher for nonsib mating compared with sib mating. We also compared our estimates of inbreeding from the experimental crosses to population genetic estimates from adults sampled from three populations in the field. Inbreeding, in terms of nonrandom mating (Keller & Waller, 2002), was rare in the field, but relatively more common in laboratory settings with controlled pairwise crosses.

Despite the potential for self‐fertilization in bryozoans, and early studies on bryozoans even suggesting that selfing was widespread and the predominant mode of sexual reproduction (citations in Silén, 1966), available evidence now suggests that outcrossing is common in bryozoans (Hoare & Hughes, 2001; Hunter & Hughes, 1993; Ryland & Bishop, 1993; Silén, 1972; Temkin, 1994; Yund & McCartney, 1994). It should also be noted, however, that the self‐fertilization rate does vary considerably within some species and negatively covaries with inbreeding depression (Hughes et al., 2009). In *Bugula stolonifera*, fertilization success per colony in colonies reared in isolation ranged from 38% and 59% (Johnson, 2010). Although selfing in *B. stolonifera* resulted in the release of viable offspring, it resulted in severe inbreeding depression after larval release (Johnson, 2010), suggesting that the relatively high inbreeding coefficients estimated from genetic markers in natural populations of this species result from dispersal limitation and biparental inbreeding (Johnson & Woollacott, 2010, 2012, see also Grosberg, 1987, 1991 for an ascidian example). In contrast, there was no marker‐based evidence for inbreeding in three natural populations of *B. neritina* in this study, similar to that found for other spermcasters with short larval dispersal distances (Bishop & Ryland, 1993; Hoare, Hughes, & Goldson, 1999). In *Celleporella hyalina*, average fertilization success per colony in colonies reared in isolation was only 1.4% (and only 25% of colonies produced embryos) and no viable offspring were produced (Hoare & Hughes, 2001), a result similar to this study and to Cancino, Hughes, and Ramirez (1991) and Hunter and Hughes (1993). Unlike most previous studies, our study also experimentally assessed biparental inbreeding and found that it was more common than selfing. In *C. hyalina* sib matings, fertilization success per colony averaged 28%–30%, but was also associated with severe inbreeding depression (Hoare & Hughes, 2001). Accordingly, inbreeding coefficients in natural populations of adults are not significantly different to zero (Hoare et al., 1999), as found here for *B. neritina* (*cf*. Johnson & Woollacott, 2012).

The differences in reproductive success between self, sib mating, and nonsib mating within populations, and the low frequency of inbreeding in the field, suggest several interesting, nonmutually exclusive, factors that determine the overall mating and dispersal strategy. First, sperm may not be released unless cues from conspecifics, or unrelated conspecifics, are detected (Bishop, Manriquez, & Hughes, 2000). This possibility remains to be tested because it was not confirmed that all colonies grown in isolation actually released sperm (except for the one colony where selfing was detected). Few studies have investigated cues for sperm release in bryozoans, but sperm release has been induced by light in some species (e.g., *Celleporella hyalina*) (Manríquez, Hughes, & Bishop, 2001). Furthermore, in *C. hyalina*, investment in male zooids depends on environmental conditions (Hunter & Hughes, 1995), rather than conspecific cues (Hughes et al., 2009), though colonies produce more male zooids at colony edges that contact a physical barrier, including the edge of the same or different colony (Hoare et al., 1999).

Second, fertilization success may be biased against self, and to a lesser extent sibling, sperm if such bias has evolved to avoid inbreeding depression. For example, females may actively reject self or sibling sperm. Sperm may actively avoid self or sibling colonies, or actively choose unrelated, or nonself, colonies, perhaps through chemotaxis (Miller, 1985). In our experiments, colonies exposed to a conspecific clearly released sperm, because genetic markers confirmed outcrossing in all replicates with two colonies. Furthermore, the production of viable juveniles positively covaried within each mating pair: if one colony produced many outcrossed offspring, its partner also produced many outcrossed offspring. These observations suggest that both self and nonself sperm were present during the same period, providing the opportunity to self‐fertilize, but that fertilization success was biased against self sperm. In the bryozoan *Electra posidoniae*, sperm appear to be functionally immobile once shed and drift into the feeding currents of lophophores (Silén, 1966). At this point, zooids could potentially reject certain sperm cells, as they can when selecting among phytoplankton cells (Okamura, 1990). When inside the tentacle crown of a lophophore, sperm suddenly become activated, performing a series of violent jerks that allows them to cling to the outer, unciliated surface of a tentacle (Silén, 1966). Multiple sperm cells can attach to a single tentacle crown. Sperm then swim to the internally located ovum, potentially guided by cues released from the egg. At this point, there may also be fertilization blocks to self, or sibling, sperm, where unrelated sperm typically win the fertilization (Grosberg, 1987).

Third, the number of viable offspring may be influenced by early inbreeding depression during the ~7‐day brooding period (i.e., after fertilization and before larval release). For example, self‐fertilization may have occurred, but resulted in all embryos being aborted within hours to days before embryos were visible in the ovicell and before larvae were released and counted. Similarly, inbreeding may have occurred in the field, but inbred individuals died before being sampled, resulting in low marker‐based estimates of inbreeding. Such inbreeding depression could be caused by lower food intake per zooid that supplies brooded inbred embryos, or inbred embryos requiring more nutrients from the maternal zooid because of suboptimal cellular biochemistry caused by the genetic effects of inbreeding (Hoare & Hughes, 2001; Hughes et al., 2009). Although we did not notice aborted embryos in the dishes or during larval release, several other studies on bryozoans have noted selfed embryos aborted during brooding (Hoare et al., 1999; Hunter & Hughes, 1993; Johnson, 2010). In our experiments, only four colonies produced more than 50 offspring from sib matings, and crosses between sibs may just reflect a smaller fraction of aborted embryos compared with colonies reared in isolation. When compared to random crosses from the same population, the number of viable juveniles produced may result from increased inbreeding depression as kinship between parents increased. The possibility of multiple larvae being produced in sequence from a single zooid from stored sperm and that the time of fertilization and stage of brooding could differ among zooids within a colony, makes it challenging to properly estimate inbreeding depression during brooding in this species. However, once larvae were released, survival of all larvae and settlers over an eight‐day period was equally high.

If *B. neritina* preferentially outcrosses, it is possible that selfing may simply occur later in life, and the total number of juveniles produced for inbred crosses would be higher had the experiment been run for longer. In freshwater snails, for example, outcrossing species delay the age at first reproduction when they do not encounter mates, whereas self‐fertilizing species reproduce without mates as soon as they are sexually mature, which is independent of mate availability (Escobar et al., 2011). This delay in selfing is consistent with theory predicting that individuals should be more reluctant to self‐fertilize their eggs and risk longer waiting times under strong inbreeding depression (Tsitrone, Duperron, & David, 2003). We found that individuals grown in the absence of another colony produced ovicells at the same time and also produced the same number of ovicells on average, as colonies mated with another colony. Such evidence suggests equal investment in female reproduction and that reproductive capability was not delayed in isolated individuals. Some plants delay selfing after prolonged periods with little outcrossing opportunity, but many also do not, possibly because certain mating systems are more or less predisposed to benefit from delayed selfing (Goodwillie & Weber, 2018). Self‐fertilization as a “emergency option” has been suggested to explain low levels of self‐fertilization in primarily outcrossing spermcasters (Bishop & Ryland, 1993; Hunter & Hughes, 1993; Yund & McCartney, 1994). However, the ability to actively capture, concentration, and store waterborne sperm from very dilute suspension in spermcasters suggests that sperm limitation may be less of an issue at low population density (Hughes et al., 2002; Pemberton et al., 2003). Sperm in spermcasting species may also be inactive during dispersal, promoting longevity and drifting over greater distances, and activated in the presence of a conspecific (Bishop, 1998; Johnson & Yund, 2004; Manríquez et al., 2001). Ultimately, whether self‐fertilization becomes more common later in life in isolated colonies remains to be tested in this system.

The lack of evidence for consistent inbreeding in the field suggests potential additional factors: the short larval dispersal that does occur (Keough & Chernoff, 1987) could already be enough to avoid outcrossing with relatives (Grosberg, 1987, 1991; Perrin & Mazalov, 2000; Ravigné et al., 2006). In other spermcasters, most fertilizations typically occur between nearest neighbors (Grosberg, 1991; Yund & McCartney, 1994), and nearest neighbors are often only centimeters away in *B. nertina* (Keough, 1989), leading to frequent competition among males (Pemberton et al., 2003; Yund, 1998). Male function may be disadvantaged by dispersal to vacant areas as a result of being outcompeted for fertilizations by other colonies that are nearer to conspecifics. However, if female function is not severely sperm limited, spreading larvae over a range of distances, including those that exceed the small spatial scale of effective sperm dispersal under male competition, would reduce potential for inbreeding (Ravigné et al., 2006). Furthermore, the typically high densities of colonies in the field, or the ability to utilize sperm from dilute suspension, provide opportunities for outcrossing with unrelated individuals and reduced need for self‐fertilization as reproductive assurance (Hughes et al., 2009). Though the scale of sperm dispersal in this system is unknown, we suspect that larval dispersal in the order of meters to tens of meters, plus the potential for adult rafting over greater distances (Keough & Chernoff, 1987), contributes to avoiding inbreeding in this system (Grosberg, 1987, 1991; Phillippi & Yund, 2017). This would also support the hypothesis that larval dispersal over much greater distances, in the order of kilometers (Shanks, 2009), in other benthic marine invertebrates does not evolve to avoid inbreeding (Burgess, Baskett, Grosberg, Morgan, & Strathmann, 2016; Strathmann, 2007). In this system, both dispersal and mate choice could explain why inbreeding was rare in the field, but relatively more common in laboratory settings with controlled pairwise crosses.

In summary, the potential for inbreeding does not inevitably lead to inbreeding. Therefore, traits that predict inbreeding potential (such as simultaneous hermaphroditism, and those that lead to kin associations after dispersal) may have a low capacity to explain observed variation in inbreeding, without a more synthetic understanding of how mating and dispersal strategies coevolve. In our study, the differences in parental reproductive success between self, sib, and nonsib crosses, and the low frequency of inbreeding in the field, suggest possible roles of early inbreeding depression, mate choice, and differential dispersal of sperm and larvae in determining the amount of inbreeding seen in these populations of *B. neritina*. Furthermore, any occasional inbreeding in these simultaneous hermaphrodites is more likely to occur from biparental inbreeding than from selfing, probably as a by‐product of needing to transfer male gametes in water currents prior to fertilization. Estimates of the selfing rate from population genetic markers would also be biased by the presence of biparental inbreeding. Ongoing work will understand the relative contribution of inbreeding depression, mate choice, and differential dispersal of sperm and larvae to the overall mating and dispersal strategy in this species, which will contribute to a broader understanding of dispersal and mating system evolution more generally.

## CONFLICT OF INTEREST

The authors declare no conflicts of interest.

## AUTHOR CONTRIBUTIONS

SCB conceived and funded the project, collected samples, performed the experiments, analyzed the data, and wrote the manuscript. LS and MB performed the experiments and collected data.

## Data Availability

Dryad: https://doi.org/10.5061/dryad.c73080n
