# Supplementary material for: How relatedness between mates influences reproductive success: An experimental analysis of self‐fertilization and biparental inbreeding in a marine bryozoan
Source: Ecol Evol. 2019 Sep 5;9(19):11353–66. doi: 10.1002/ece3.5636 (PMC6802076; doi:10.1002/ece3.5636)
Supplement: Supplementary file 1 [file ECE3-9-11353-s001.docx]

**Supporting Information for:**

**Scott C. Burgess, Lisa Sander, and Marília Bueno. How relatedness between mates influences reproductive success: an experimental analysis of self-fertilization and biparental inbreeding in a marine bryozoan. *Ecology and Evolution***

**Appendix S1. Isolation and characterization of 16 microsatellite loci in the bryozoan *Bugula neritina*.**

Here we describe the isolation and characterization of 16 new polymorphic microsatellite loci for the arborescent bryozoan *Bugula neritina* (Linnaeus, 1978) (Phylum: Bryozoa; Class: Gymnolaemata; Order: Cheilostomata). The eight grandparents used in the progeny arrays (four from Dog Island and four from Marine Lab, described in main article), plus an additional four samples from Dog Island and seven samples from Saint Joseph Bay collected in 2016 (*unpublished data*; all samples from the Florida Panhandle coastline) all had identical sequences of the mitochondrial cytochrome oxidase c subunit I (COI), which were also identical to the Type S1 haplotype of Davidson and Haygood (1999) (GenBank accession numbers AF061432, AF061426) and the Type S (Shallow) haplotype of McGovern and Hellberg (2003) (GenBank accession number AY173425). This, plus sampling in the same region by others (Davidson and Haygood 1999; McGovern and Hellberg 2003; Mackie et al. 2006; Fehlauer-Ale et al. 2014), suggests that all samples analyzed here were the Type S haplotype.

*Microsatellite library development*

Total genomic DNA was extracted from ~30mg of tissue using OMEGA Bio-Tek E.Z.N.A® Tissue DNA Kit following the manufacturer’s protocol. DNA quality and quantity were assessed by spectroscopy (NanoDrop 1000). DNA from three colonies were pooled and sent to the Evolutionary Genetics Core Facility (EGCF) at Cornell University for construction of a genomic DNA library for the isolation and Illumina/MiSeq sequencing of DNA fragments enriched for microsatellite loci. Library construction and screening followed EGCF protocols. Briefly, genomic DNA was digested with 4-base restriction enzymes that generated blunt-ended fragments. After adenylation, Illumina TruSeq adapters were ligated to the digested DNA and the resulting fragments were enriched for microsatellites by hybridization to, and magnetic capture of, biotinylated repeat probes (representing two unique dimers, five unique trimers, seven unique tetramers and two unique pentamers). Enriched genomic fragments were amplified and barcoded by PCR. Libraries with unique barcodes were pooled and sequences were generated with Illumina/MiSeq reagents, protocols, and hardware (2 x 250 bp paired reads). Barcode-sorted reads were quality-trimmed, scanned for Illumina adaptor sequences, and assembled with NGen/SeqMan Pro software (DNASTAR). Consensus files were exported as fasta files and microsatellite repeats and associated genotyping primers were summarized with msatcommander software. 1,497 unique microsatellite loci and primer pairs were obtained. Minimum consecutive perfect repeat lengths were at least six (12 bp) for any dimer and at least five for any trimer, tetramer, or pentamer.

*PCR amplification and screening*

96 primer pairs were screened for amplification success across 8 samples. Candidate primer pairs were chosen based on read counts ≥10 and <250 and repeat counts ≥5. Di- and tri-nucleotide repeat motifs were not considered. All oligos were synthesized by Eurofins Genomics, Louisville KY. All PCRs to assess amplification were performed in an 8ul volume using 1-10ng of template DNA, 0.25uM each primer, 0.01% BSA, and 2X GoTaq® Green Master Mix. PCR thermal cycling parameters were as follows: initial denaturation at 95° (5 minutes) followed by 35 cycles of 95 (30 seconds), 56° (30 seconds), 72° (45 seconds) and a final extension at 72° (10 minutes). Amplification products were then visualized on a 1.5% agarose gel with GelRed.

*Assessing polymorphism*

Of 96 potential markers screened, 41 showed amplification across all samples tested. These 41 primer pairs were moved forward to be assessed for polymorphism via fragment analysis on an Applied Biosystems 3730 Genetic Analyzer. All forward primers were then tailed at the 5’ end with one of the following universal tails: (M13) 5’-TGT AAA ACG ACG GCC AGT-3’, (C) 5’-CAG GAC CAG GCT ACC GTG-3’, or (D) 5’-CGG AGA GCC GAG AGG TG-3’. One of the following fluorescent dyes was then incorporated into each of the final amplicons via a second step PCR containing an oligo homologous to the previously described tails: FAM, HEX, NED, or PET. Primer mixes were made containing 0.5uM Forward primer with tail, 2uM Reverse primer, and 2uM fluorescent primer, in TE buffer. PCR was carried out as above except with 2X GoTaq® Colorless Master Mix. PCR thermal cycling parameters were as follows: initial denaturation at 95° (5 minutes) followed by 8 cycles of 95° (30 seconds), 56° (30 seconds), 72° (45 seconds) and 30 cycles of 95° (30 seconds), 53° (30 seconds), 72° (45 seconds) and a final extension at 72° (15 minutes). For each sample, amplicons were pooled in approximately equal ratios with 5 amplicons per pool. Samples for fragment analysis contained 1.5μl of pooled amplicons, 0.15μl LIZ size standard and 12ul Hi-Di formamide. Purified PCR products were separated on an Applied Biosystems 3730 Genetic Analyzer with Capillary Electrophoresis in the Biology Core Facility at Florida State University. Fragment sizes were assessed using the program Geneious ver. 9.1.8 and all alleles were called manually. 16 microsatellite loci were found to amplify consistently and proved suitable for downstream analyses (Table S1).

The number of alleles per locus, observed and expected heterozygosities, and deviations from Hardy-Weinberg equilibrium (F_IS_) were assessed in the program GenoDive v2.0b23 (Meirmans and Van Tienderen 2004). Polymorphic Information Content and the estimated frequency of null alleles was calculated in the program Cervus v3.0.7 (Kalinowski et al. 2007). The sequential Bonferroni correction was utilized to adjust significance levels compensating for multiple comparisons within the same test. Since it is difficult to identify null alleles with certainty in the absence of known parent-offspring relationships, we utilized the progeny array to determine marker mistyping rates based on observed mismatches between known parents and offspring in the program Cervus v3.0.7. Finally, we assessed multi-locus linkage disequilibrium by calculating the index of association (I_A_) and the standardized index of association that accounts for the number of loci ($\bar{r}_{d}$) using the *poppr* package in R (Kamvar et al. 2014). The null hypothesis tested was that alleles observed at different loci are not linked and alleles recombine freely into new genotypes.

*Description of loci characteristics*

Samples of 74 adult colonies, randomly collected from three sites, were genotyped at 16 microsatellite loci. These samples served as the grandmother generation, and eight individuals served as grandparents in the progeny array. Details on the progeny array can be found in the main text. Across all populations, the number of alleles per locus ranged from 3-17. All loci were found to be polymorphic, with no evidence for significant deviations from Hardy-Weinberg equilibrium (Table S2-S4).

In the progeny array, there were 5 mother-offspring mismatches and the mistyping rate ranged from 0 (which occurred at 14 loci) to 0.036 (Table S5). All five mismatches could be explained by the inheritance of a null allele (i.e. when offspring and known mother are homozygous for different alleles). Mismatches occurred in three families at two loci (Table S6).

**Table S1:** Microsatellite loci and primers developed for *Bugula neritina* (Type S)*.*

| Locus | GenBank Accession no. | Primer Sequence (5’ – 3’) | Expected allele size |
| --- | --- | --- | --- |
| B.ner1 | MN329814 | F: GCACTATATAAGGGGTGTTTCG  R: CATGCCTAATGAATACCCTGG | 266 – 294 |
| B.ner2 | MN329815 | F: GTGCAATTTGTTACACTGTCCAC  R: GCACTACAGAGGAGTTTGAGTTG | 347 – 411 |
| B.ner3 | MN329816 | F: TGTCAGAATTGTGTTTGTATGGAC  R: GGGAGGTCACAATGCTAGTTTG | 213 – 226 |
| B.ner4 | MN329817 | F: TGTGTAGACAGTGTGCAGCC  R: CAGTGGACCAAGACATAACCAG | 262 – 363 |
| B.ner5 | MN329818 | F: TCCTCTGGTCTCCACTTCTTTAG  R: TTCATGTGTTGTCTCCTTCTGG | 203 – 272 |
| B.ner6 | MN329819 | F: AGTAGAGAAGTCCCACAGCATG  R: CCTTCCACACTCACGAGCTC | 255 – 502^a^ |
| B.ner7 | MN329820 | F: TGTGCTCAAGGTAAAGGGATAC  R: GTGATGCTGTTTGTCCGAATTG | 225 – 250 |
| B.ner8 | MN329821 | F: TACCATGCTTCTGTTAACACGC  R: TTAGTGAGGGTATGATGCAGGC | 174 – 303 |
| B.ner9 | MN329822 | F: TAAATTTCTGCAGCACCGTCTG  R: ACCGTCTACATACTCTTGACCC | 231 – 297 |
| B.ner10 | MN329823 | F: TACTCAACACCCTTATCCTGCC  R: GTTGGGTCCCTCTTCAGATTATC | 321 – 366 |
| B.ner11 | MN329824 | F: GGCCTAACTAGAACTAAGCACG  R: AGGCATTCGATATAGTAGGCTG | 135 – 169 |
| B.ner12 | MN329825 | F: TCCACCTTCATTCAATAGCACC  R: TTGAATGTTGTACCAGCACACC | 302 – 357 |
| B.ner13 | MN329826 | F: TCAACAACTTCACGTGGCTTTG  R: AACCAAACAACATGCCTCAGAG | 187 – 265 |
| B.ner14 | MN329827 | F: ATTCTTGTGCCTACTACTGCTG  R: ACTTGTGGTTGTTTCTTGTTTCC | 273 – 333 |
| B.ner15 | MN329828 | F: GGAAAGAGTCACTGCCTATAACC  R: GATTTCCAGAGTCAAGTTTGGAG | 198 – 220 |
| B.ner16 | MN329829 | F: ACACTTATGAGGACCTTGGCTC  R: TTATGACCGACACAGTTGATCG | 376 – 499 |

^a^459 – 502 most common.

**Table S2:** Characteristics of microsatellite loci at Site 1 (Dog Island) for 30 randomly sampled adults from the field. H_o_ = observed heterozygosity, H_e_ = expected heterozygosity, F_IS_, PIC = Polymorphic Information Content, F(Null) = estimated frequency of null alleles. Significant deviations of F_IS_, from zero denoted by *(P < 0.003 = 0.05/16).

| Locus | Number of individuals | Number of alleles | H_o_ | H_e_ | F_IS_ | PIC | F(Null) |
| --- | --- | --- | --- | --- | --- | --- | --- |
| Bner1 | 29 | 6 | 0.828 | 0.818 | -0.012 | 0.775 | -0.0145 |
| Bner2 | 30 | 7 | 0.767 | 0.802 | 0.044 | 0.756 | 0.0153 |
| Bner3 | 30 | 13 | 0.700 | 0.741 | 0.055 | 0.703 | 0.0064 |
| Bner4 | 30 | 17 | 0.967 | 0.916 | -0.056 | 0.893 | -0.0354 |
| Bner5 | 30 | 7 | 0.733 | 0.857 | 0.145 | 0.821 | 0.0657 |
| Bner6 | 29 | 6 | 0.552 | 0.650 | 0.152 | 0.578 | 0.0860 |
| Bner7 | 30 | 11 | 0.667 | 0.667 | 0.001 | 0.630 | 0.0093 |
| Bner8 | 30 | 14 | 0.767 | 0.876 | 0.125 | 0.845 | 0.0529 |
| Bner9 | 30 | 9 | 0.867 | 0.845 | -0.025 | 0.811 | -0.0217 |
| Bner10 | 30 | 4 | 0.733 | 0.668 | -0.097 | 0.587 | -0.0496 |
| Bner11 | 30 | 8 | 0.733 | 0.733 | 0.000 | 0.684 | -0.0152 |
| Bner12 | 30 | 3 | 0.533 | 0.503 | -0.061 | 0.440 | -0.0621 |
| Bner13 | 30 | 7 | 0.667 | 0.639 | -0.044 | 0.595 | -0.0158 |
| Bner14 | 30 | 7 | 0.700 | 0.761 | 0.081 | 0.707 | 0.0309 |
| Bner15 | 30 | 5 | 0.800 | 0.747 | -0.072 | 0.694 | -0.0376 |
| Bner16 | 30 | 8 | 0.700 | 0.734 | 0.046 | 0.675 | 0.0173 |

**Table S3:** Characteristics of microsatellite loci at Site 2 (Marine Lab) for 10 randomly sampled adults from the field. H_o_ = observed heterozygosity, H_e_ = expected heterozygosity, F_IS_, PIC = Polymorphic Information Content, F(Null) = estimated frequency of null alleles. Significant deviations of F_IS_, from zero denoted by *(P < 0.003 = 0.05/16).

| Locus | Number of individuals | Number of alleles | H_o_ | H_e_ | F_IS_ | PIC | F(Null) |
| --- | --- | --- | --- | --- | --- | --- | --- |
| Bner1 | 10 | 6 | 0.800 | 0.800 | 0.000 | 0.730 | -0.0142 |
| Bner2 | 10 | 7 | 0.600 | 0.844 | 0.289 | 0.763 | 0.1576 |
| Bner3 | 10 | 8 | 0.900 | 0.833 | -0.080 | 0.770 | -0.0811 |
| Bner4 | 10 | 11 | 0.900 | 0.917 | 0.018 | 0.858 | -0.0258 |
| Bner5 | 10 | 9 | 0.900 | 0.878 | -0.025 | 0.815 | -0.0418 |
| Bner6 | 10 | 6 | 0.800 | 0.700 | -0.143 | 0.628 | -0.0923 |
| Bner7 | 10 | 5 | 0.900 | 0.739 | -0.218 | 0.670 | -0.1300 |
| Bner8 | 10 | 9 | 0.900 | 0.833 | -0.080 | 0.769 | -0.0681 |
| Bner9 | 10 | 8 | 0.800 | 0.789 | -0.014 | 0.729 | -0.0276 |
| Bner10 | 10 | 5 | 0.800 | 0.722 | -0.108 | 0.635 | -0.0774 |
| Bner11 | 10 | 6 | 0.900 | 0.811 | -0.110 | 0.741 | -0.0784 |
| Bner12 | 10 | 4 | 0.400 | 0.617 | 0.351 | 0.526 | 0.2126 |
| Bner13 | 10 | 4 | 0.500 | 0.539 | 0.072 | 0.452 | -0.0116 |
| Bner14 | 10 | 5 | 0.900 | 0.756 | -0.191 | 0.674 | -0.1128 |
| Bner15 | 10 | 4 | 0.700 | 0.606 | -0.156 | 0.535 | -0.1012 |
| Bner16 | 10 | 5 | 0.800 | 0.711 | -0.125 | 0.627 | -0.0972 |

**Table S4:** Characteristics of microsatellite loci at Site 3 (One More Time) for 34 randomly sampled adults from the field. H_o_ = observed heterozygosity, H_e_ = expected heterozygosity, F_IS_, PIC = Polymorphic Information Content, F(Null) = estimated frequency of null alleles. Significant deviations of F_IS_, from zero denoted by *(P < 0.003 = 0.05/16).

| Locus | Number of individuals | Number of alleles | H_o_ | H_e_ | F_IS_  (p-value) | PIC | F(Null) |
| --- | --- | --- | --- | --- | --- | --- | --- |
| Bner1 | 34 | 6 | 0.794 | 0.774 | -0.026 | 0.727 | -0.0215 |
| Bner2 | 34 | 9 | 0.618 | 0.746 | 0.172 | 0.693 | 0.0854 |
| Bner3 | 34 | 11 | 0.765 | 0.767 | 0.003 | 0.727 | -0.0108 |
| Bner4 | 34 | 16 | 0.882 | 0.909 | 0.029 | 0.886 | 0.0061 |
| Bner5 | 34 | 8 | 0.765 | 0.814 | 0.060 | 0.777 | 0.0169 |
| Bner6 | 34 | 6 | 0.735 | 0.644 | -0.142 | 0.593 | -0.0853 |
| Bner7 | 34 | 8 | 0.794 | 0.741 | -0.072 | 0.684 | -0.0459 |
| Bner8 | 34 | 13 | 0.735 | 0.793 | 0.073 | 0.762 | 0.0374 |
| Bner9 | 34 | 13 | 0.794 | 0.790 | -0.006 | 0.751 | -0.0064 |
| Bner10 | 34 | 8 | 0.882 | 0.696 | -0.268 | 0.638 | -0.1371 |
| Bner11 | 34 | 7 | 0.765 | 0.720 | -0.062 | 0.660 | -0.0407 |
| Bner12 | 34 | 4 | 0.500 | 0.581 | 0.140 | 0.491 | 0.0700 |
| Bner13 | 34 | 8 | 0.500 | 0.485 | -0.031 | 0.435 | 0.0061 |
| Bner14 | 34 | 8 | 0.706 | 0.713 | 0.010 | 0.661 | -0.0023 |
| Bner15 | 34 | 5 | 0.824 | 0.712 | -0.157 | 0.646 | -0.0824 |
| Bner16 | 34 | 8 | 0.824 | 0.677 | -0.217 | 0.620 | -0.1157 |

**Table S5:** Mistyping rates estimated from analysis of the progeny array using the program Cervus v3.0. Column headings are as follow: number of known parent-offspring pairs compared for the locus (N compared), number of known parent-offspring pairs with no alleles in common at the locus. (N mismatching), number of known parent-offspring pairs mismatching where known parent and offspring are both homozygous (N null) (such mismatches could have been caused by inheritance of a null allele), average probability of detecting a mismatch (Detection probability), and the estimated rate of mistyping, calculated as the ratio of the number of mismatches to the number compared scaled by the average probability of detecting a mismatch (Mistyping rate). The mean mistyping rate across all loci was 0.0034.

| Locus | N compared | N mismatching | N null | Detection probability | Mistyping rate |
| --- | --- | --- | --- | --- | --- |
| Bner1 | 189 | 0 | 0 | 0.3993 | 0 |
| Bner2 | 193 | 0 | 0 | 0.3660 | 0 |
| Bner3 | 193 | 0 | 0 | 0.4280 | 0 |
| Bner4 | 193 | 0 | 0 | 0.5316 | 0 |
| Bner5 | 188 | 0 | 0 | 0.5097 | 0 |
| Bner6 | 193 | 3 | 3 | 0.2162 | 0.03594434 |
| Bner7 | 192 | 0 | 0 | 0.3824 | 0 |
| Bner8 | 192 | 0 | 0 | 0.6074 | 0 |
| Bner9 | 193 | 0 | 0 | 0.5235 | 0 |
| Bner10 | 193 | 0 | 0 | 0.2111 | 0 |
| Bner11 | 183 | 2 | 2 | 0.3083 | 0.01772318 |
| Bner12 | 193 | 0 | 0 | 0.2123 | 0 |
| Bner13 | 193 | 0 | 0 | 0.1722 | 0 |
| Bner14 | 192 | 0 | 0 | 0.3839 | 0 |
| Bner15 | 193 | 0 | 0 | 0.2700 | 0 |
| Bner16 | 170 | 0 | 0 | 0.3227 | 0 |

**Table S6:** Mismatches between offspring and known maternal genotypes. If offspring and known mother are homozygous for different alleles, a ‘Yes’ is shown in the column labelled ‘Null?’, indicating that the mismatch could be explained by the inheritance of a null allele. Mother ID 36.6 was part of a between-population cross that is not included in the main results section of the paper.

| Locus | Mother ID (Genotype) | Offspring ID (Genotype) | Null? |
| --- | --- | --- | --- |
| Bner6 | 30.8 (459/459) | 30.8.2 (463/463) | Yes |
|  |  | 30.8.21 (463/463) | Yes |
|  |  | 30.8.23 (463/463) | Yes |
| Bner11 | 36.6 (135/135) | 36.6.2 (165/165) | Yes |
|  | 36.8 (147/147) | 36.8.2 (165/165) | Yes |

**Literature cited**

Davidson, S. K., and M. G. Haygood. 1999. Identification of sibling species of the bryozoan *Bugula neritina* that produce different anticancer bryostatins and harbor distinct strains of the bacterial symbiont “*Candidatus Endobugula sertula*.” Biol. Bull. 196:273–280.

Fehlauer-Ale, K. H., G. E. Lim-Fong, E. Ale, M. R. Pie, A. Waeschenbach, and J. A. Mackie. 2014. Cryptic species in the cosmopolitan *Bugula neritina* complex (Bryozoa, Cheilostomata). Zool. Scr. 43:193–205.

Kalinowski, S. T., M. L. Taper, and T. C. Marhsall. 2007. Revising how the computer program CERVUS accommodates genotyping error increases success in paternity assignment. Mol. Ecol. 16:1099–1106.

Kamvar, Z. N., J. F. Tabima, and N. J. Grünwald. 2014. *Poppr* : an R package for genetic analysis of populations with clonal, partially clonal, and/or sexual reproduction. PeerJ 2:e281. PeerJ Inc.

Mackie, J. A., M. J. Keough, and L. Christidis. 2006. Invasion patterns inferred from cytochrome oxidase I sequences in three bryozoans, *Bugula neritina*, *Watersipora subtorquata*, and *Watersipora arcuata*. Mar. Biol. 149:285–295. Springer-Verlag.

McGovern, T. M., and M. E. Hellberg. 2003. Cryptic species, cryptic endosymbionts, and geographic variation in chemical defenses in the bryozoan *Bugula neritina*. Mol. Ecol. 12:1207–1215.

Meirmans, P. G., and P. H. Van Tienderen. 2004. GENOTYPE and GENODIVE: two programs for the analysis of genetic diversity of asexual organisms. Mol. Ecol. Notes 4:792–794.
